# Ecological drivers of the colon cancer microbiome: evidence for niche-based selection over stochastic colonization

**DOI:** 10.3389/fonc.2026.1888804

**Published:** 2026-10-06

**Authors:** Wei Su, Zhanshan (Sam) Ma

**Affiliations:** 1 Department of Radiology, Affiliated Hospital of Yunnan University, Kunming, China; 2 Microbiome Medicine and Advanced AI Technology, Kunming, China; 3 Computational Biology and Medical Ecology Lab, Kunming Institute of Zoology, Chinese Academy of Sciences, Kunming, China

**Keywords:** colon cancer, neutral theory, niche theory, normal adjacent tissue (NT), primary tumor (PT), tumor microenvironment (TME), tumor tissue microbiome

## Abstract

Colon cancer is a leading global malignancy influenced by genetic, environmental, and microbial factors. This study integrates ecological theories, including Hubbell’s neutral theory, augmented by Ning et al.’s (2019, PNAS) stochasticity analysis framework, and classic niche theory, to assess the contributions of stochastic colonization and deterministic niche selection in shaping microbiome assembly and dynamics in colon cancer tissues. Specialist species were identified using Levins’ niche breadth analysis. By reanalyzing the datasets from Nejman et al., including 22 normal adjacent tissue (NT) and 22 primary tumor (PT) samples, we find: (i) deterministic niche selection appears to dominate community assembly and diversity maintenance in both NT and PT microbiomes, as demonstrated by the normalized stochasticity ratio (NSR: 0.267 for NT, 0.287 for PT; both below 0.5; Wilcoxon PT>NT, p=0.027). Only 18.2% NT and 27.3% PT samples fit the neutral model, confirming stochastic drift plays a limited role and niche selection predominates in structuring tissue microbiomes. (ii) Through niche breadth analysis, we identified 106 specialist species, of which 14 are known CRC-associated taxa, including *Fusobacterium nucleatum*, *Escherichia coli*, *Enterococcus faecalis*, and *Peptostreptococcus anaerobius*. These species have established roles in inflammation, genotoxicity, immune modulation, and chemoresistance. (iii) These findings suggest that deterministic niche-based selection, rather than stochastic colonization, appears to govern microbial community assembly in colon cancer tissues. (iv) We postulate that the tumor microenvironment imposes biochemical filters—including hypoxia, lactate accumulation, and altered pH—that select for specialist species capable of adapting to these conditions. (v) While the sample size is modest and validation in independent cohorts is needed, our study demonstrates an integrative framework for understanding colon cancer microbiome dynamics. We propose testable hypotheses for future research: (i) specialist species abundance may correlate with tumor stage or patient survival; (2) depletion of specialists may alter tumor progression in preclinical models; and (3) hybrid neutral-niche models may better fit larger longitudinal datasets. By integrating ecological theory with microbiome research, this study offers insights into the forces driving microbial community assembly in tumor tissues and highlights potential targets for microbiome-based diagnostics and therapeutics.

## Introduction

Colorectal cancer (CRC) remains one of the most prevalent and deadly malignancies worldwide, driven by a complex interplay of genetic, environmental, and microbial factors. Among these, the gut microbiota has emerged as an active participant in tumor initiation, progression, and treatment response, particularly within the densely microbial colon environment (1). The gut microbiome—comprising bacteria, viruses, fungi, and archaea—maintains intestinal homeostasis, yet dysbiosis is strongly implicated in CRC. This imbalance typically involves an expansion of pro-inflammatory bacteria and a depletion of beneficial butyrate producers, triggering chronic inflammation, DNA damage, and oncogenic pathway activation (2–5). Mechanistically, CRC-associated microbes act through several converging pathways. Genotoxic species, such as pks+ *E. coli* (producing colibactin) and enterotoxigenic *Bacteroides fragilis* (ETBF), directly damage host DNA and activate Wnt/β-catenin signaling to drive proliferation (6). Concurrently, pro-inflammatory taxa like *Fusobacterium nucleatum* potentiate tumorigenesis via NF-κB activation and immune cell recruitment (7, 8). A distinct *F. nucleatum* clade has been shown to dominate the colorectal cancer niche (9). Metabolic influences further shape the tumor microenvironment: while microbial-derived short-chain fatty acids (e.g., butyrate) exert anti-carcinogenic effects, secondary bile acids produced by other bacteria promote malignancy. Biofilms in right-sided CRC disrupt the mucosal barrier and promote inflammation (10). Key bacteria act distinctly: *F. nucleatum* promotes inflammation and chemoresistance (11), ETBF activates Wnt/β-catenin, and pks+ *E. coli* induces DNA damage via colibactin.

The microbiome also holds promise as a biomarker for CRC screening, diagnosis, and prognosis, with microbial signatures (e.g., *F. nucleatum* or pks+ *E. coli*) potentially identifying high-risk individuals (12). Furthermore, the microbiome influences treatment efficacy: certain bacteria metabolize chemotherapy drugs, reducing effectiveness, while others enhance immunotherapy response by modulating host immunity (13). Therapeutic strategies targeting the microbiome—including fecal microbiota transplantation (FMT), probiotics, prebiotics, engineered bacteria, and bacteriocins—offer emerging avenues for CRC prevention and treatment (14). Despite these advances, challenges remain. The low microbial biomass in tumor tissues complicates differentiation between resident microbiota and contaminants (15), and the complexity of microbial interactions hinders therapy development. Quantitative profiling has revealed that microbiome confounders can challenge predicted microbial targets in CRC development (16). Future research should establish causal relationships, standardize diagnostics, integrate microbiome-based therapies into personalized treatment, and investigate early-onset CRC (EOCRC), which may exhibit distinct biological features compared to late-onset disease (17, 18).

Yet, the ecological rules governing microbial community assembly in the context of cancer remain unclear—specifically, whether deterministic (niche-based) or stochastic (neutral) processes dominate. Classical niche theory (19) posits that each species occupies a distinct niche, while neutral models (20) assume ecological equivalence and predict community structure driven by stochastic birth, death, and immigration. These frameworks have been applied to human and environmental microbiomes (21–26), including studies in other cancers. In colorectal cancer, Dai et al. (27) found that only ~4% of gut (stool not tissue) microbiome samples fit neutral predictions, concluding deterministic niche differentiation dominates in both healthy and CRC patients. In lung cancer, Mei et al. (28) reported deterministic tumor-driven selection in tumor tissues, contrasting with stochastic assembly in normal adjacent tissue. In breast tissue, Li et al. (21) observed neutral species decline from 84.8% to 75.2% during malignant transformation. These findings support the view that tumor microenvironments impose selective pressures across cancer types, though direct comparisons with tissue-resident CRC microbiomes remain limited. Recent atlas-level studies have revealed coordinated interactions between tumor, immune, and microbial compartments in colon cancer (29), and host genetics have been shown to interact with intratumoral microbiota in CRC progression (30).

To resolve the balance between stochastic and deterministic assembly in colon cancer, this study applies Hubbell’s neutral theory (20), Ning et al.’s (31) NSR framework, Levins’ niche breadth (32), and classic niche theory (33) to colon cancer microbiome data (34). Specifically, we aim to (i) quantify the degree of neutrality in colon-associated microbial communities, (ii) identify compositional shifts between normal and tumor tissues, and (iii) elucidate the ecological mechanisms—particularly niche-based selection over stochastic colonization—governing community assembly in tumor tissues. We hypothesize that the tumor microenvironment exerts strong niche-based selection, favoring deterministic over stochastic assembly. By integrating neutral and niche frameworks, this study offers a promising ecological perspective on colon microbiome dynamics, with implications for microbiome-based diagnostics and therapeutics.

## Methods and datasets

### Cancer tissue microbiome datasets

The datasets utilized in this study were originally published by Nejman et al. (34), who conducted a comprehensive analysis of approximately 2000 tumors and their adjacent normal tissues across eight cancer types: breast, lung, ovary, pancreas, melanoma, bone, colon, and brain tumors. The methodology for processing these samples is described in detail by Nejman et al. (34). For this study, the colon microbiome dataset was selected for analysis (**Supplementary Table 1**), which included microbiome samples from both primary tumor (PT) and normal tissue (NT). A total of 44 samples (22 NT and 22 PT) were analyzed.

### Normalized stochasticity ratio

To quantify ecological stochasticity, the framework proposed by Ning et al. (31) was employed. This mathematical framework is based on the principle that deterministic processes can either increase or decrease community similarity relative to null expectations. The stochasticity ratio (ST) ranges from 0 to 1, where 0 indicates entirely deterministic community assembly and 1 indicates entirely stochastic assembly. However, when expected stochasticity is low, ST may overestimate stochasticity. To address this limitation, ST is normalized to yield the Normalized Stochasticity Ratio (NSR), which has been demonstrated to provide higher accuracy and precision compared to ST (31). Following Ning et al. (31), NSR values range from 0 to 1 with 50% (0.5) as the boundary point: values below 0.5 indicate deterministic dominance, while values above 0.5 indicate stochastic dominance. This threshold was applied consistently across all comparisons between NT and PT microbiomes.

### Hubbell’s neutral theory of biodiversity

Hubbell’s (20) neutral theory has become a widely applied framework for understanding the assembly and diversity of microbial communities (27, 35–37). Hubbell’s theory is an individual-based sampling model that provides a mechanistic explanation for species abundance distributions (SAD) in ecological communities. It posits that all individuals in a saturated local community are ecologically equivalent, meaning they share identical birth, death, and migration rates, aside from random fluctuations (20). This assumption allows for the prediction of community structure based on stochastic processes rather than species-specific traits or environmental adaptations (20, 35–37).

In this study, Etienne’s (38, 39) sampling formulae and the “Exact” test of neutrality were employed to evaluate the fit of Hubbell’s neutral theory to the colon cancer microbiome data. These statistical procedures involve fitting the neutral model to observed species abundance distributions (SAD) and comparing the model predictions to the actual data, represented by operational taxonomic unit (OTU) tables or other microbiome datasets. Unlike species sorting analyses, which incorporate environmental factors (explanatory matrix X), neutrality tests assume that species responses to environmental gradients are neutral, meaning they do not differ significantly across species. This assumption simplifies the analysis by focusing solely on stochastic processes.

Etienne’s “Exact” neutrality test is based on sequential construction schemes and does not require an alternative model for hypothesis testing. This is a significant advantage, as it avoids the complexities associated with validating alternative SAD models, such as lognormal, log-series, or power law distributions (39). The computational procedures for implementing the neutrality test, including maximum likelihood estimation (MLE) and log-likelihood ratio (LLR) tests, are detailed in Etienne (22, 38, 39). The code used for testing the neutral theory in this study was sourced from the supplementary information provided by Etienne (38).

### Microbial niche breadth and overlap

Niche theory is a fundamental concept in ecology with a long-standing history (19). It defines a niche as an area (or volume) in a multidimensional space formed by the relationship between species and environmental properties. In microbiology, niche theory is used to describe how environmental properties (for non-host microbiomes), host-microbe interactions, and species-species interactions determine the distribution of microorganisms (37). From a quantitative perspective, Levins’ niche breadth (BN) (32) is widely applied in ecological studies.

Levins’ niche breadth (BN) is calculated as:


$$Levins^{\prime }\;B_{N}=\frac{1}{R\sum_{i=1}^{R}p_{i}^{2}}$$

where *R* is the number of resources states, *p_i_* represents the relative use of the *i-*th resources. If a species utilizes all resources equally, *B_N_* approaches 1 and this species is considered a generalist with a broad, non-discriminatory niche (40). By contrast, if a species’ B_N_ approaches 1/*R*, it is considered a specialist with a narrow, discriminatory niche. In this study, R was set to 2, representing the two tissue types compared (NT and PT). For each species, *p_i_* corresponds to its relative abundance in each tissue type, normalized across the two states. This binary framework directly addresses our central question of whether tumorigenesis imposes selective pressure that alters niche breadth between normal and tumor tissues.

Levins’ niche overlap (LO) pair wisely compares the proportional use of resource *r* by species *i* and species *j*:


$$LO_{i,j}=\frac{\sum_{i,j=1}(p_{ir})(p_{jr})}{\sum_{i=1}(p_{ir}^{2})}$$

For microbiome datasets, *p_j_* and *p_j_* ​ represent the proportional abundance of species *i* and *j*, respectively, and *r*_i_ represents the proportional value of any given environmental variable. R is considered the sum of environments.

To classify species as specialists or generalists, we calculated p-values and FDR-adjusted p-values (Benjamini-Hochberg). To avoid false positives due to low or sparse abundance, we applied a limit of quantification (LOQ), below which a species is considered undetectable with 95% certainty. Following Finn et al. (33), the LOQ is species-specific, derived from each taxon’s null abundance distribution across samples. Thus, a species with consistently low but reproducible abundance may exceed its LOQ, while a species with highly sporadic counts may not. Specialists were defined as species with B_N_ close to 0.5, p-adjusted < 0.05, and abundance above the LOQ. In this study, we used Finn et al. (33) computational code, and the LOQ values for each species are computed automatically with the program.

## Results

### NSR-based stochasticity in normal vs. tumor microbiome assembly

We first sought to quantify the relative contributions of deterministic and stochastic processes in the assembly of normal tissue microbiome (NTM) and primary tumor microbiome (PTM) communities. To achieve this, we employed the Normalized Stochasticity Ratio (NSR) framework for each dataset (31). The NSR values for NTM communities (0.267) were lower than those for PTM communities (0.287), with a Wilcoxon test indicating PT > NT (p = 0.027) (**Supplementary Table 2**). However, when considering the boundary point, the NSR values for both NTM and PTM communities were below 0.5, indicating that deterministic processes play a more dominant role in the assembly of both NTM and PTM communities.

### Hubbell’s neutral theory of biodiversity

Next we apply Hubbell’s neutral theory, specifically the exact neutrality tests using Etienne’s sampling formula, for evaluating the stochasticity *vs.* selection in the tissue microbiomes of colon cancer and the healthy control. The test results are exhibited in **Supplementary Tables 3**, **S4**. Among 22 NT samples, only four (18.2%) adhered to the predictions of the neutral model, while six out of 22 PT samples (27.3%) aligned with neutrality. This low level of adherence suggests that stochastic processes alone cannot fully account for microbial community assembly in colon cancer microbiomes. The majority of samples showed significant deviations from neutrality, with observed log-likelihood values (Log(L0)) falling outside the 95% confidence intervals predicted by the neutral model.

**Figure 1** presents the rank abundance curves for representative neutrality-adherent samples in a square configuration (two NT and two PT shown; remaining six omitted to conserve space). In the figure, the solid red lines represent the observed species abundance data, while the black dashed lines depict the simulated datasets generated under the neutral model. The X-axis represents the species rank order in abundance (from most to least abundant), and the Y-axis represents the natural logarithm of species abundance.

**Figure 1:**
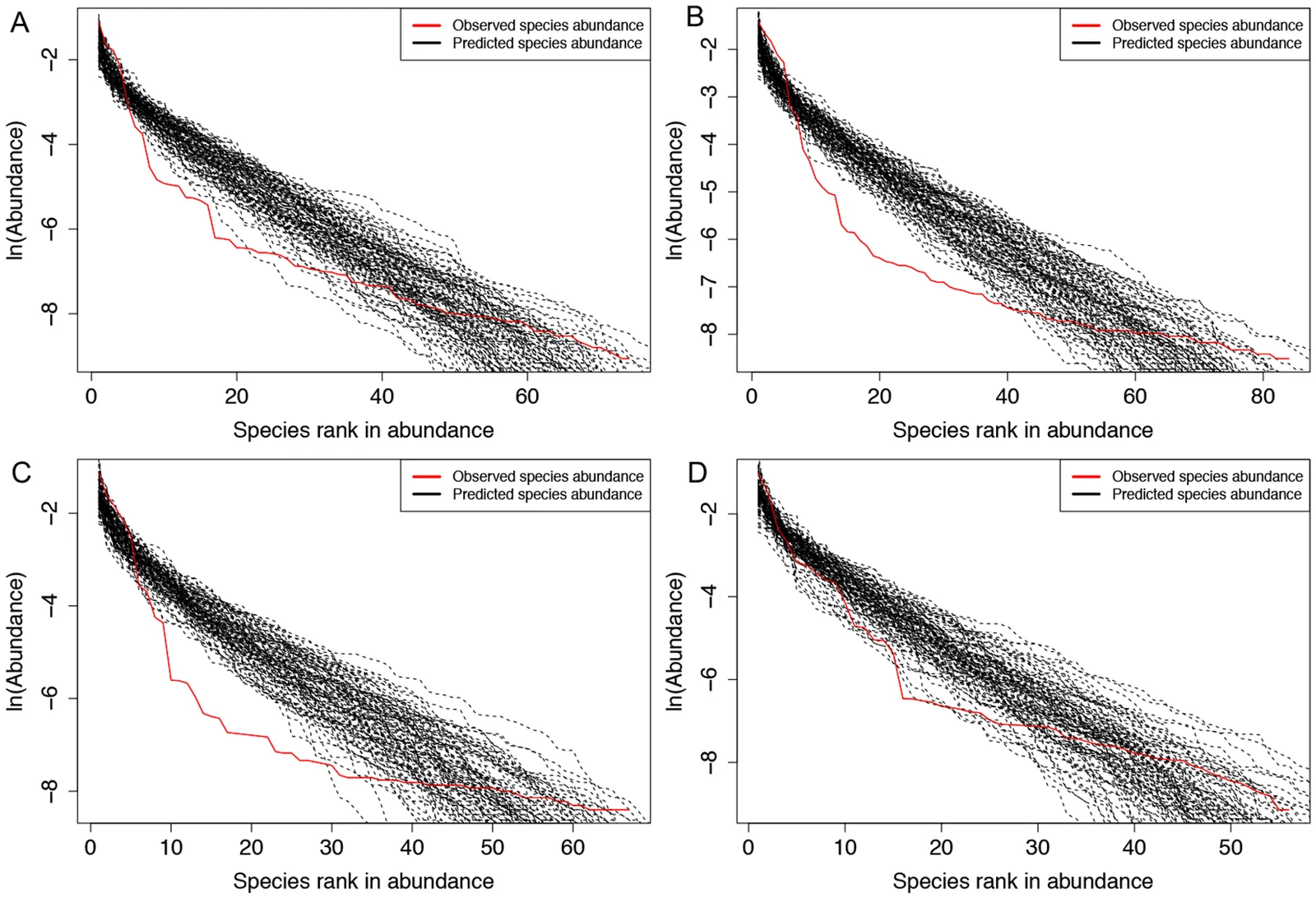
Rank abundance curves for neutrality-adherent samples: The rank abundance curves for four samples that align well with the neutrality model are depicted in the figure. In the plot, the solid red lines represent the observed species abundance data, while the black dashed lines represent the simulated datasets generated under the neutral model. The X-axis indicates the species rank order in abundance (from most to least abundant), and the Y-axis represents the natural logarithm of species abundance. Samples **(A, B)** are derived from normal tissue (NT), while samples **(C, D)** are from primary tumor (PT).

### Niche breadth and overlap for generalist species

As demonstrated above, the neutral theory models—including the neutral community model and Hubbell’s neutral theory of biodiversity—along with the Normalized Stochasticity Ratio (NSR), indicate that stochastic processes alone cannot fully explain the assembly of microbial communities in normal tissue microbiomes (NTM) and primary tumor microbiomes (PTM) of colon cancer. Therefore, deterministic processes are likely to play a more significant role in shaping these microbial communities. This suggests that factors such as host-specific conditions (e.g., immune response, tumor microenvironment) and environmental filtering (e.g., pH, oxygen levels) may exert a stronger influence on microbial community assembly in colon cancer than random, stochastic processes. These factors may also shape the prevalence and distribution of generalist species in both NTM and PTM, further underscoring the importance of deterministic drivers in microbial community dynamics.

In this study, Levins’ niche breadth and overlap were employed to identify specialist species and explore significant species interactions. **Supplementary Table 5** highlights specialist species characterized by a niche breadth (B_N_) value close to 0.5 (where 1/R, with R = 2), a p-adjusted value < 0.05, and abundances not below the limit of quantification (LOQ). These specialist species may arise due to various deterministic factors—such as host-specific conditions (e.g., immune response, tumor microenvironment) or environmental filtering (e.g., pH, oxygen levels)—that differentiate normal tissue (NTM) from primary tumor tissue (PTM) in colon cancer. Their presence emphasizes the potential influence of deterministic processes in shaping microbial community assembly and interactions in these distinct tissue types.

Among the 106 specialist species identified, several are known CRC-associated taxa. **Table 1** lists the top CRC-associated specialist species, including *Fusobacterium nucleatum* (B_N_ = 0.604), *Fusobacterium gonidiaformans* (0.624), *Bacteroides xylanisolvens* (0.579), *Escherichia coli* (0.583), *Enterococcus faecalis* (0.567), *Clostridium perfringens* (0.509), *Peptostreptococcus anaerobius* (0.500), *Campylobacter ureolyticus* (0.501), *Streptococcus infantis* (0.618), *Streptococcus oralis* (0.562), *Abiotrophia defectiva* (0.513), *Gemella morbillorum* (0.516), *Brachyspira aalborgi* (0.500), and *Sarcina ventriculi* (0.500). These species have established roles in CRC pathogenesis, including inflammation, genotoxicity, immune modulation, and chemoresistance.

**Table 1:** Top CRC-associated specialist species identified by Levins’ niche breadth analysis*.

Species	Levins’ B_N_	Known CRC mechanism	Supporting references
*Fusobacterium nucleatum*	0.604	Inflammation, NF-κB activation, chemoresistance, poor prognosis	(7, 11, 41)
*Fusobacterium gonidiaformans*	0.624	Oral-gut translocation, CRC enrichment	(41)
*Bacteroides xylanisolvens*	0.579	Immune modulation, CRC-associated	(2)
*Escherichia coli* (pks+)	0.583	Colibactin genotoxicity, DNA damage	(2, 42)
*Enterococcus faecalis*	0.567	Inflammation, oxidative DNA damage	(6)
*Clostridium perfringens*	0.509	Toxin production, enteritis, CRC association	(4)
*Peptostreptococcus anaerobius*	0.500	CRC progression, inflammation	(7)
*Campylobacter ureolyticus*	0.501	Mucosal disruption, CRC enrichment	(11)
*Streptococcus infantis*	0.618	CRC enrichment, SBSEC member	(12)
*Streptococcus oralis*	0.562	CRC enrichment, biofilms, immune evasion	(12)
*Abiotrophia defectiva*	0.513	Opportunistic pathogen, CRC association	(7)
*Gemella morbillorum*	0.516	Opportunistic pathogen, CRC enrichment	(11)
*Brachyspira aalborgi*	0.500	Intestinal spirochete, CRC	(10)
*Sarcina ventriculi*	0.500	Gastric/intestinal, CRC association	(5)

*All species met specialist criteria: Levins’ B_N_ close to 0.5 (R = 2), *p*.adj < 0.05 (FDR-corrected), abundance above LOQ (limit of quantification). Full list of 106 specialists are documented in **Supplementary Table 5**.

The combined relative abundance of specialist species exceeds 0.50 (50%) in some samples, as shown in **Figure 2**, despite the fact that only 106 specialist species were identified out of more than 10,000 total species in the NT and PT cohorts. This finding highlights the ecological dominance of a relatively small number of specialist species in structuring microbial communities. Interestingly, most samples defined by Hubbell’s neutrality model exhibit lower relative abundances of specialist species compared to other samples. These include Hiseq5RDB3, Hiseq5RDB33, Hiseq5RDB39, Hiseq5RDB8, Hiseq5RDB34, Hiseq5RDB4, Hiseq5RDB41, and Hiseq5RDB65. However, Hiseq5RDB55 is an exception, displaying a high relative abundance of specialist species. This pattern suggests that while stochastic processes (as described by Hubbell’s neutrality model) may dominate in some samples, deterministic factors—such as host-specific conditions or environmental filtering—likely play a significant role in driving the high abundance of specialist species in others. Additionally, some samples with lower abundances of specialist species appear to be influenced by negative deterministic factors related to their habitat. In the case of this study, the most important host-specific condition is obviously the development and progression of colon cancer.

**Figure 2:**
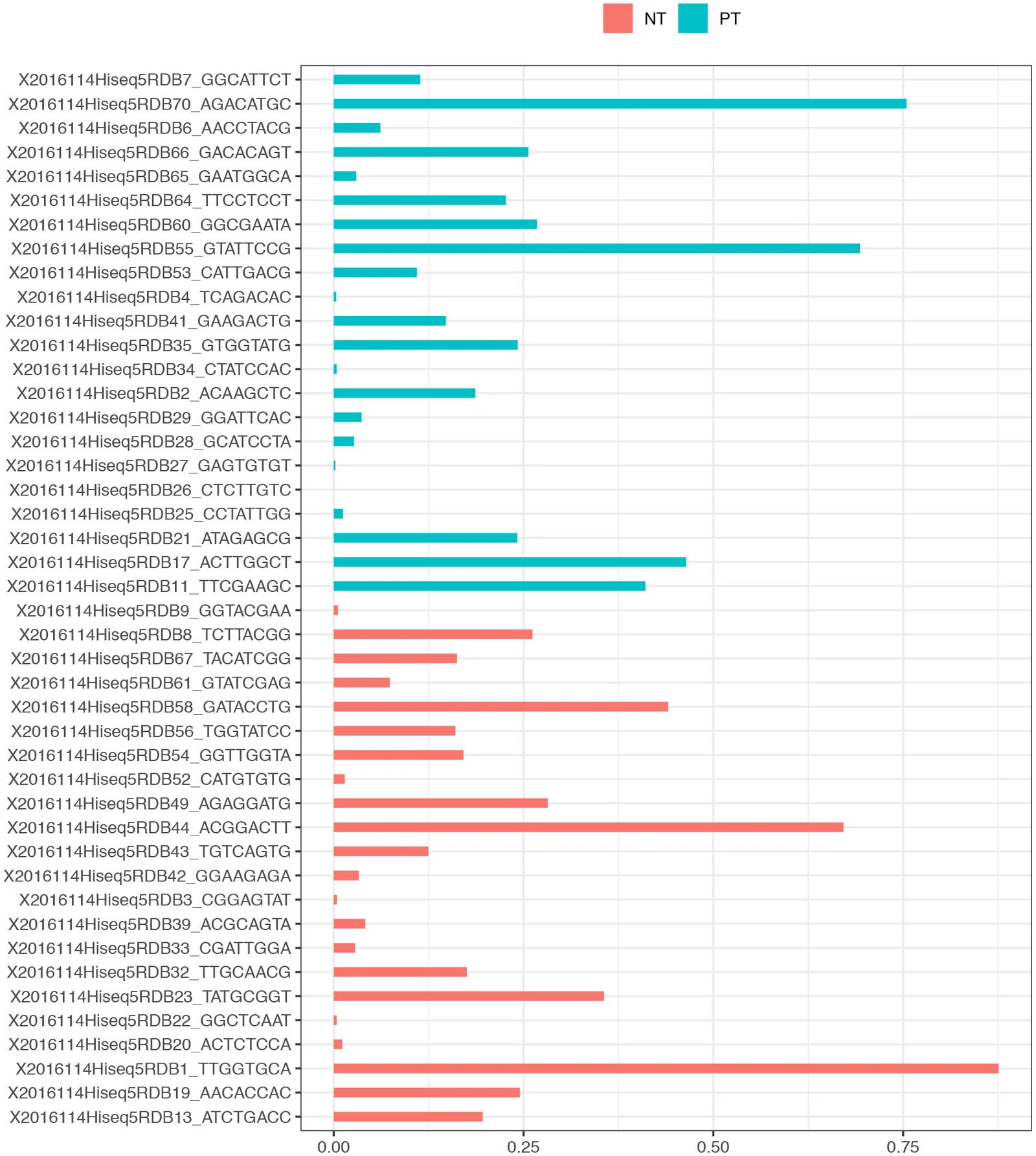
Cumulative relative abundance of specialist species across samples. The X-axis represents cumulative relative abundance of specialist species; the Y-axis denotes individual samples. This visualization highlights the ecological dominance of specialist species in shaping microbial community structure across different samples.

## Discussion and conclusions

### Discussion

This study provides insights into the ecological dynamics of the colon cancer microbiome, highlighting the interplay between deterministic and stochastic processes in microbial community assembly. By applying Hubbell’s neutral theory and classic niche theory, our results suggest that deterministic niche selection—such as host-specific conditions and environmental filtering—play a substantial role in structuring microbial communities in both normal tissue (NT) and primary tumor (PT) environments. This is supported by the low adherence to the neutral model (only 10/44 samples) and NSR values below 0.3, indicating that stochastic processes alone cannot fully explain community assembly. However, we acknowledge that unmodeled stochasticity (e.g., speciation, dispersal) may still contribute. Nevertheless, deterministic selection forces appear to play a dominant role in structuring colon microbiomes, a finding consistent with observations in lung cancer (28).

A key finding is the identification of specialist species that dominate microbial communities in colon cancer tissues. These species, characterized by narrow niche breadth and high relative abundance, appear to be influenced by deterministic factors such as the tumor microenvironment, immune response, and local conditions. Their dominance suggests they may shape community structure and potentially contribute to tumorigenesis through inflammation, immune modulation, and metabolic interactions. Specifically, we identified 14 CRC-associated specialist species (**Table 1**) with known roles in CRC pathogenesis, including *Fusobacterium nucleatum* (inflammation, chemoresistance), *Escherichia coli* (colibactin genotoxicity), *Enterococcus faecalis* (oxidative DNA damage), and *Peptostreptococcus anaerobius* (immune evasion). A distinct *F. nucleatum* clade has been shown to dominate the colorectal cancer niche, supporting the ecological significance of this species in CRC (9). This aligns with previous studies emphasizing host-microbe interactions and environmental pressures in shaping the gut microbiome in health and disease (5, 6). The differences in community assembly between NT and PT tissues further underscore the impact of the tumor microenvironment, suggesting that the TME creates selective pressures favoring certain taxa.

The tumor microenvironment imposes specific biochemical filters that drive deterministic selection. Key factors include hypoxia (selecting for facultative anaerobes such as *E. coli* and *F. nucleatum*), lactate accumulation (favoring species capable of metabolizing alternative carbon sources), altered pH (restricting acid-sensitive taxa), and immune infiltration (selecting for immunomodulatory species). These conditions collectively create a niche environment that favors specialist species capable of adapting to multiple stressors, providing a mechanistic basis for the deterministic dominance observed in our study.

The low proportion of neutrality-adherent samples (18.2% NT, 27.3% PT) aligns with prior work questioning the applicability of neutral theory in complex microbial ecosystems, particularly in disease contexts (22). Deviations from neutrality suggest that niche-based processes—competition for resources and host-microbe interactions—are more influential in colon microbiomes.

One additional piece of evidence supporting the previous conclusion is the failure of the Sloan near-neutral model (35, 36) to fit the colon microbiome datasets. As shown in **Supplementary Table 2**, the model fitting failed in all three comparisons, with R² values below zero, indicating poor model performance. This failure further confirms the dominant role of niche selection in shaping colon tissue microbiomes.

Several limitations should be acknowledged. First, the sample size (*n* = 22 per group) is modest, and lack of an independent validation cohort limits generalizability. Second, the low microbial biomass in tumor tissues poses challenges for profiling, though we used the Nejman et al. (34) dataset, which addressed contamination rigorously. Third, the complexity of microbial interactions within the TME warrants further investigation. Fourth, analysis, while comprehensive, still does not fully account for all stochastic processes such as speciation and dispersal. Fifth, the small sample size precluded application of more sophisticated hybrid neutral-niche models (24), which we suggest as a future direction when larger datasets become available. Sixth, while we discuss the ecological implications of our findings, the current study is primarily computational and does not directly measure biochemical factors of the tumor microenvironment—such as lactate accumulation, hypoxia, and altered pH—that may act as deterministic niche filters shaping the microbial community. Future work integrating metabolomic or physiological data with microbial profiling would be valuable to address this gap.

### Conclusion

This study demonstrates that deterministic niche-based selection appears to dominate microbial community assembly in colon cancer, with stochastic processes playing a limited role. A small set of specialist species—including known CRC-associated taxa such as *F. nucleatum*, *E. coli*, and *E. faecalis*—drives community structure in both normal and tumor tissues. These findings provide an ecological framework for interpreting tumor-microbiome interactions and identify candidate specialist species for further mechanistic and translational study.

Future work should test three hypotheses: (i) specialist species abundance correlates with tumor stage or patient survival; (ii) depletion of specialists alters tumor progression in murine models; and (iii) hybrid neutral-niche models better fit larger longitudinal cohorts. By integrating ecological theory with microbiome research, we can advance our understanding of host-microbe interactions in colon cancer and inform the development of microbiome-based diagnostics and therapeutics.

## Data Availability

The datasets reanalyzed in this study was originally published by Nejman et al. (2020).
