# Supplementary material for: Ecological drivers of the colon cancer microbiome: evidence for niche-based selection over stochastic colonization
Source: Front Oncol. 2026 Oct 6;16:1888804. doi: 10.3389/fonc.2026.1888804 (PMC13680166; doi:10.3389/fonc.2026.1888804)
Supplement: Supplementary file 1 [file DataSheet1.pdf]

Online Supplementary Tables for: Su & Ma (2026) Ecological Drivers of the Colon Cancer Microbiome: Evidence for Niche-Based Selection Over Stochastic Colonization

Table S1. A brief description of colon cancer microbiome datasets (Nejman et al 2020)

**Cancer Disease**	**Groups**	**Number of Samples**	**Number of Species**	**Number of Reads**	**Mean of Reads**
Colon	NT	22	527	1173655	53348
	PT	22	482	1082104	49187

(NT = Normal adjacent tissue; PT = Primary tumor)

Table S2. The means of the normalized stochasticity ratio (*NSR*) for each Cancer Microbiome dataset

**Disease**	**Normalized Stochasticity Ratio (NSR)**		**Wilcoxon test for the NSR**		
	**NT**	**PT**	≠	>	<
Colon	0.267	0.287	0.055	0.973	0.027

Table S3. Exact neutrality test results for the normal tissue (NT) microbiome using Etienne's sampling formula.

**Sample ID**	***J***	***S***	***θ***	***m***	**Log(L_0)**	**Log(L_1)**	**95% (Low)**	**Neutral (?)**
X2016114Hiseq5RDB1_TTGGTGCA	71613	27	4.862	0.003	-158.943	-147.7	-151.199	No
X2016114Hiseq5RDB13_ATCTGACC	168435	244	46.759	0.011	-705.634	-653.465	-657.416	No
X2016114Hiseq5RDB19_AACACCAC	20076	18	5.452	0.001	-98.858	-90.822	-93.212	No
X2016114Hiseq5RDB20_ACTCTCCA	52649	210	40.34	0.04	-459.147	-425.417	-427.72	No
X2016114Hiseq5RDB22_GGCTCAAT	64438	388	73.77	0.077	-587.702	-537.245	-538.95	No
X2016114Hiseq5RDB23_TATGCGGT	75165	225	40.009	0.047	-502.11	-481.275	-483.981	No
X2016114Hiseq5RDB3_CGGAGTAT	85987	74	9.952	0.059	-251.91	-277.735	-281.445	Yes
X2016114Hiseq5RDB32_TTGCAACG	7612	10	2.888	0.002	-55.283	-47.769	-49.513	No
X2016114Hiseq5RDB33_CGATTGGA	49735	84	12.11	0.089	-242.79	-264.232	-267.344	Yes
X2016114Hiseq5RDB39_ACGCAGTA	70590	167	26.85	0.067	-405.103	-414.07	-416.896	Yes
X2016114Hiseq5RDB42_GGAAGAGA	78878	97	17.667	0.012	-352.139	-344.874	-347.959	No
X2016114Hiseq5RDB43_TGTCAGTG	93129	123	25.425	0.007	-451.865	-408.692	-412.124	No
X2016114Hiseq5RDB44_ACGGACTT	10934	22	6.163	0.003	-111.334	-92.904	-94.983	No
X2016114Hiseq5RDB49_AGAGGATG	16493	24	4.869	0.007	-116.371	-109.21	-111.492	No
X2016114Hiseq5RDB52_CATGTGTG	36929	286	53.86	0.119	-431.016	-401.578	-403.147	No
X2016114Hiseq5RDB54_GGTTGGTA	68995	53	10.49	0.004	-244.529	-236.637	-239.889	No
X2016114Hiseq5RDB56_TGGTATCC	71826	59	12.319	0.004	-281.893	-259.193	-262.212	No
X2016114Hiseq5RDB58_GATACCTG	877	21	6.196	0.063	-53.197	-48.422	-49.194	No
X2016114Hiseq5RDB61_GTATCGAG	15135	79	16.831	0.034	-209.314	-202.435	-204.34	No

X2016114Hiseq5RDB67_TACATCGG	39278	219	38.769	0.114	-384.949	-379.953	-382.186	No
X2016114Hiseq5RDB8_TCTTACGG	27577	131	23.179	0.085	-287.146	-286.436	-288.514	Yes
X2016114Hiseq5RDB9_GGTACGAA	47310	298	58.323	0.072	-463.458	-449.252	-451.081	No

J: the total number of reads in the sample,
S: the number of species in the sample,
θ: the fundamental biodiversity,
m: immigration probability,
Log(L_0): the log-likelihood of the observed sample,
Log(L_1): the mean log-likelihood predicted by the neutral model,
95% (Low): the low limit of 95% confidence interval of the log-likelihood from the 100 predicted neutral model.

Table S4. Exact neutrality test results for the primary tumor (PT) microbiome using Etienne's sampling formula.

Sample ID	*J*	*S*	θ	*m*	Log(L_0)	Log(L_1)	95% (Low)	Neutral (?)
X2016114Hiseq5RDB11_TTCGAAGC	67912	128	19.631	0.066	-342.495	-362.628	-365.958	YES
X2016114Hiseq5RDB17_ACTTGGCT	95432	56	11.969	0.002	-277.153	-263.394	-267.472	NO
X2016114Hiseq5RDB2_ACAAGCTC	7473	10	2.839	0.002	-52.576	-48.685	-50.51	
X2016114Hiseq5RDB21_ATAGAGCG	14498	47	8.863	0.033	-157.972	-153.402	-155.63	
X2016114Hiseq5RDB25_CCTATTGG	49572	210	39.822	0.048	-452.656	-414.445	-416.916	
X2016114Hiseq5RDB26_CTCTTGTC	603	14	5.02	0.032	-42.267	-34.67	-35.388	
X2016114Hiseq5RDB27_GAGTGTGT	62880	273	49.516	0.062	-509.431	-484.289	-486.501	
X2016114Hiseq5RDB28_GCATCCTA	30760	155	26.721	0.111	-320.077	-312.458	-314.717	
X2016114Hiseq5RDB29_GGATTCAC	78560	417	78.072	0.068	-658.856	-586.309	-588.175	
X2016114Hiseq5RDB34_CTATCCAC	44491	67	9.216	0.111	-201.76	-224.809	-228.06	YES
X2016114Hiseq5RDB35_GTGGTATG	22408	49	11.411	0.007	-184.118	-179.248	-181.502	NO
X2016114Hiseq5RDB4_TCAGACAC	93568	56	7.521	0.039	-222.419	-238.997	-243.062	YES
X2016114Hiseq5RDB41_GAAGACTG	93671	120	16.958	0.076	-334.167	-367.532	-371.546	YES
X2016114Hiseq5RDB53_CATTGACG	33223	253	52.511	0.067	-416.795	-380.649	-382.223	NO
X2016114Hiseq5RDB55_GTATTCCG	50049	142	23.459	0.07	-312.791	-353.862	-356.632	YES
X2016114Hiseq5RDB6_AACCTACG	114420	115	23.441	0.006	-452.582	-417.206	-421.406	NO
X2016114Hiseq5RDB60_GGCGAATA	75235	82	17.912	0.004	-343.735	-313.885	-317.069	
X2016114Hiseq5RDB64_TTCCTCCT	35452	94	17.657	0.027	-288.702	-274.733	-277.449	
X2016114Hiseq5RDB65_GAATGGCA	22276	43	6.293	0.089	-134.168	-150.822	-153.434	YES
X2016114Hiseq5RDB66_GACACAGT	535	14	6.399	0.023	-35.661	-34.646	-35.344	NO
X2016114Hiseq5RDB7_GGCATTCT	41931	268	52.857	0.07	-463.266	-418.256	-420.005	
X2016114Hiseq5RDB70_AGACATGC	47184	40	7.286	0.007	-191.609	-179.841	-183.286	

J: the total number of reads in the sample,
S: the number of species in the sample,
θ: the fundamental biodiversity,
m: immigration probability,
Log(L_0): the log-likelihood of the observed sample,
Log(L_1): the mean log-likelihood predicted by the neutral model,
95% (Low): the low limit of 95% confidence interval of the log-likelihood from the 100 predicted neutral model.

Table S5. Levins' niche breadth (B_N) results for each species in normal and primary tumor tissues of colon cancer, filtered by *P*.adj < 0.05 (FDR-adjusted) and abundance above the limit of quantification (LOQ).

Species	Levins's B_N	*P-Value*	*P.adj*	Below LOQ (?)
Acidobacteria_Acidobacteriia_Acidobacteriales_Acidobacteriaceae_Unknown.genus10_Unknown.species3	0.500	0.000	0.001	N
Actinobacteria_Actinobacteria_Acidimicrobiales_Acidimicrobiaceae_Ilumatobacter_Unknown.species12	0.500	0.000	0.001	N
Actinobacteria_Actinobacteria_Actinomycetales_Corynebacteriaceae_Corynebacterium_Unknown.species1740	0.500	0.000	0.001	N
Actinobacteria_Actinobacteria_Actinomycetales_Corynebacteriaceae_Corynebacterium_Unknown.species846	0.500	0.000	0.001	N
Actinobacteria_Actinobacteria_Actinomycetales_Intrasporangiaceae_Ornithinimicrobium_Unknown.species14	0.500	0.000	0.001	N
Actinobacteria_Actinobacteria_Actinomycetales_Microbacteriaceae_Microbacterium_Unknown.species73	0.500	0.000	0.001	N
Bacteroidetes_Bacteroidia_Bacteroidales_Bacteroidaceae_Bacteroides_Unknown.species10	0.500	0.000	0.001	N
Bacteroidetes_Bacteroidia_Bacteroidales_Bacteroidaceae_Bacteroides_Unknown.species1382	0.500	0.000	0.001	N
Bacteroidetes_Bacteroidia_Bacteroidales_Bacteroidaceae_Bacteroides_Unknown.species1669	0.500	0.000	0.001	N
Bacteroidetes_Bacteroidia_Bacteroidales_Bacteroidaceae_Bacteroides_Unknown.species746	0.500	0.000	0.001	N
Bacteroidetes_Bacteroidia_Bacteroidales_Bacteroidaceae_Bacteroides_Bacteroides.xylanisolvens	0.579	0.007	0.011	N
Bacteroidetes_Bacteroidia_Bacteroidales_Porphyromonadaceae_Barnesiella_Unknown.species1047	0.524	0.001	0.002	N
Bacteroidetes_Bacteroidia_Bacteroidales_Porphyromonadaceae_Barnesiella_Unknown.species1062	0.504	0.000	0.001	N
Bacteroidetes_Bacteroidia_Bacteroidales_Porphyromonadaceae_Parabacteroides_Unknown.species5	0.500	0.000	0.001	N
Bacteroidetes_Bacteroidia_Bacteroidales_Porphyromonadaceae_Porphyromonas_Porphyromonas.asaccharolytica	0.500	0.000	0.001	N
Bacteroidetes_Bacteroidia_Bacteroidales_Prevotellaceae_Prevotella_Unknown.species1341	0.535	0.002	0.002	N
Bacteroidetes_Bacteroidia_Bacteroidales_Prevotellaceae_Prevotella_Prevotella.timonensis	0.500	0.000	0.001	N
Bacteroidetes_Bacteroidia_Bacteroidales_Unknown.family_Unknown.genus1586_Unknown.species1	0.500	0.000	0.001	N
Bacteroidetes_Flavobacteriia_Flavobacteriales_Weeksellaceae_Chryseobacterium_Unknown.species274	0.500	0.000	0.001	N
Bacteroidetes_Sphingobacteriia_Sphingobacteriales_Sphingobacteriaceae_Mucilaginibacter_Unknown.species130	0.500	0.000	0.001	N
Cyanobacteria_Chloroplast_Streptophyta_Unknown.family_Unknown.genus39_Unknown.species1	0.504	0.000	0.001	N
Cyanobacteria_ML635J.21_Unknown.order_Unknown.family_Unknown.genus42_Unknown.species1	0.500	0.000	0.001	N
Firmicutes_Bacilli_Bacillales_Bacillaceae_Bacillus_Bacillus.thermoamylovorans	0.525	0.001	0.002	N
Firmicutes_Bacilli_Bacillales_Bacillaceae.2_Tuberibacillus_Unknown.species1	0.500	0.000	0.001	N
Firmicutes_Bacilli_Bacillales_Bacillaceae.2_Tuberibacillus_Unknown.species3	0.500	0.000	0.001	N
Firmicutes_Bacilli_Bacillales_Unknown.species_Gemella_Gemella.morbillorum	0.516	0.001	0.001	N
Firmicutes_Bacilli_Gemellales_Gemellaceae_Gemella_Unknown.species147	0.589	0.009	0.014	N
Firmicutes_Bacilli_Gemellales_Gemellaceae_Gemella_Unknown.species231	0.504	0.000	0.001	N
Firmicutes_Bacilli_Gemellales_Gemellaceae_Gemella_Unknown.species313	0.507	0.001	0.001	N
Firmicutes_Bacilli_Lactobacillales_Aerococcaceae_Abiotrophia_Abiotrophia.defectiva	0.513	0.001	0.001	N
Firmicutes_Bacilli_Lactobacillales_Enterococcaceae_Enterococcus_Enterococcus.durans	0.604	0.015	0.022	N
Firmicutes_Bacilli_Lactobacillales_Enterococcaceae_Enterococcus_Enterococcus.faecalis	0.567	0.005	0.007	N
Firmicutes_Bacilli_Lactobacillales_Lactobacillaceae_Lactobacillus_Lactobacillus.zeae	0.509	0.001	0.001	N
Firmicutes_Bacilli_Lactobacillales_Streptococcaceae_Streptococcus_Unknown.species1432	0.507	0.001	0.001	N
Firmicutes_Bacilli_Lactobacillales_Streptococcaceae_Streptococcus_Unknown.species1911	0.578	0.007	0.010	N
Firmicutes_Bacilli_Lactobacillales_Streptococcaceae_Streptococcus_Unknown.species1957	0.501	0.000	0.001	N
Firmicutes_Bacilli_Lactobacillales_Streptococcaceae_Streptococcus_Unknown.species1988	0.500	0.000	0.001	N
Firmicutes_Bacilli_Lactobacillales_Streptococcaceae_Streptococcus_Unknown.species243	0.521	0.001	0.001	N

Firmicutes_Bacilli_Lactobacillales_Streptococcaceae_Streptococcus_Unknown.species341	0.546	0.002	0.004	N
Firmicutes_Bacilli_Lactobacillales_Streptococcaceae_Streptococcus_Unknown.species36	0.533	0.001	0.002	N
Firmicutes_Bacilli_Lactobacillales_Streptococcaceae_Streptococcus_Enterococcus.faecium	0.609	0.017	0.026	N
Firmicutes_Bacilli_Lactobacillales_Streptococcaceae_Streptococcus_Streptococcus.infantis	0.618	0.021	0.032	N
Firmicutes_Bacilli_Lactobacillales_Streptococcaceae_Streptococcus_Streptococcus.oralis	0.562	0.004	0.006	N
Firmicutes_Clostridia_Clostridiales_Clostridiaceae_Clostridium_Clostridium.perfringens	0.509	0.001	0.001	N
Firmicutes_Clostridia_Clostridiales_Clostridiaceae_Sarcina_Unknown.species119	0.503	0.000	0.001	N
Firmicutes_Clostridia_Clostridiales_Clostridiaceae_Sarcina_Unknown.species13	0.502	0.000	0.001	N
Firmicutes_Clostridia_Clostridiales_Clostridiaceae_Sarcina_Unknown.species134	0.500	0.000	0.001	N
Firmicutes_Clostridia_Clostridiales_Clostridiaceae.1_Sarcina_Sarcina.ventriculi	0.500	0.000	0.001	N
Firmicutes_Clostridia_Clostridiales_Lachnospiraceae_Unknown.genus3134_Unknown.species18	0.500	0.000	0.001	N
Firmicutes_Clostridia_Clostridiales_Lachnospiraceae_Unknown.genus3332_Unknown.species11	0.500	0.000	0.001	N
Firmicutes_Clostridia_Clostridiales_Lachnospiraceae_Unknown.genus80_Unknown.species1	0.505	0.000	0.001	N
Firmicutes_Clostridia_Clostridiales_Lachnospiraceae_Catonella_Unknown.species52	0.500	0.000	0.001	N
Firmicutes_Clostridia_Clostridiales_Lachnospiraceae_Coprococcus_Unknown.species1115	0.579	0.007	0.011	N
Firmicutes_Clostridia_Clostridiales_Lachnospiraceae_Coprococcus_Unknown.species64	0.500	0.000	0.001	N
Firmicutes_Clostridia_Clostridiales_Lachnospiraceae_Moryella_Moryella.indoligenes	0.500	0.000	0.001	N
Firmicutes_Clostridia_Clostridiales_Lachnospiraceae_Roseburia_Roseburia.intestinalis	0.518	0.001	0.001	N
Firmicutes_Clostridia_Clostridiales_Peptostreptococcaceae_Clostridium.XI_Unknown.species333	0.572	0.005	0.009	N
Firmicutes_Clostridia_Clostridiales_Peptostreptococcaceae_Clostridium.XI_Unknown.species544	0.500	0.000	0.001	N
Firmicutes_Clostridia_Clostridiales_Peptostreptococcaceae_Peptostreptococcus_Peptostreptococcus.anaerobius	0.500	0.000	0.001	N
Firmicutes_Clostridia_Clostridiales_Ruminococcaceae_Unknown.genus2416_Unknown.species2	0.500	0.000	0.001	N
Firmicutes_Clostridia_Clostridiales_Ruminococcaceae_Faecalibacterium_Unknown.species22	0.534	0.001	0.002	N
Firmicutes_Clostridia_Clostridiales_Ruminococcaceae_Gemmiger_Unknown.species161	0.500	0.000	0.001	N
Firmicutes_Clostridia_Clostridiales_Ruminococcaceae_Gemmiger_Unknown.species309	0.500	0.000	0.001	N
Firmicutes_Clostridia_Clostridiales_Ruminococcaceae_Gemmiger_Unknown.species316	0.500	0.000	0.001	N
Firmicutes_Clostridia_Clostridiales_Ruminococcaceae_Gemmiger_Unknown.species346	0.515	0.001	0.001	N
Firmicutes_Clostridia_Clostridiales_Ruminococcaceae_Oscillospira_Unknown.species917	0.566	0.004	0.007	N
Firmicutes_Clostridia_Clostridiales_Veillonellaceae_Dialister_Unknown.species18	0.500	0.000	0.001	N
Firmicutes_Clostridia_Clostridiales_Veillonellaceae_Megamonas_Unknown.species29	0.584	0.008	0.012	N
Firmicutes_Clostridia_Clostridiales_Veillonellaceae_Megasphaera_Unknown.species99	0.500	0.000	0.001	N
Firmicutes_Clostridia_Clostridiales_Veillonellaceae_Schwartzia_Unknown.species27	0.504	0.000	0.001	N
Firmicutes_Clostridia_Clostridiales_Veillonellaceae_Selenomonas_Unknown.species216	0.500	0.000	0.001	N
Firmicutes_Clostridia_Clostridiales_Veillonellaceae_Selenomonas_Unknown.species229	0.500	0.000	0.001	N
Firmicutes_Clostridia_Clostridiales_Veillonellaceae_Veillonella_Unknown.species105	0.500	0.000	0.001	N
Firmicutes_Clostridia_Clostridiales_Veillonellaceae_Veillonella_Unknown.species47	0.510	0.001	0.001	N
Firmicutes_Erysipelotrichi_Erysipelotrichales_Erysipelotrichaceae_Bulleidia_Unknown.species12	0.523	0.001	0.002	N
Firmicutes_Erysipelotrichia_Erysipelotrichales_Erysipelotrichaceae_Catenibacterium_Catenibacterium.mitsuokai	0.593	0.010	0.016	N
Fusobacteria_Fusobacteriia_Fusobacteriales_Fusobacteriaceae_Fusobacterium_Fusobacterium.gonidiaformans	0.624	0.025	0.038	N
Fusobacteria_Fusobacteriia_Fusobacteriales_Fusobacteriaceae_Fusobacterium_Fusobacterium.nucleatum	0.604	0.014	0.022	N
Fusobacteria_Fusobacteriia_Fusobacteriales_Leptotrichiaceae_Leptotrichia_Unknown.species236	0.500	0.000	0.001	N
Fusobacteria_Fusobacteriia_Fusobacteriales_Leptotrichiaceae_Leptotrichia_Leptotrichia.trevisanii	0.568	0.005	0.007	N

Ignavibacteriae_Ignavibacteria_Ignavibacteriales_Ignavibacteriaceae_Ignavibacterium_Unknown.species229	0.500	0.000	0.001	N
Proteobacteria_Alphaproteobacteria_Caulobacterales_Caulobacteraceae_Brevundimonas_Brevundimonas.mediterranea	0.500	0.000	0.001	N
Proteobacteria_Alphaproteobacteria_Rhizobiales_Bradyrhizobiaceae_Afipia_Unknown.species8	0.528	0.001	0.002	N
Proteobacteria_Alphaproteobacteria_Rhizobiales_Methylobacteriaceae_Methylobacterium_Methylobacterium.komagatae	0.531	0.001	0.002	N
Proteobacteria_Betaproteobacteria_Burkholderiales_Alcaligenaceae_Sutterella_Unknown.species221	0.500	0.000	0.001	N
Proteobacteria_Betaproteobacteria_Burkholderiales_Alcaligenaceae_Sutterella_Unknown.species83	0.500	0.000	0.001	N
Proteobacteria_Betaproteobacteria_Burkholderiales_Comamonadaceae_Curvibacter_Curvibacter.gracilis	0.500	0.000	0.001	N
Proteobacteria_Betaproteobacteria_Neisseriales_Neisseriaceae_Eikenella_Eikenella.corrodens	0.604	0.014	0.022	N
Proteobacteria_Epsilonproteobacteria_Campylobacterales_Campylobacteraceae_Campylobacter_Campylobacter.ureolyticus	0.501	0.000	0.001	N
Proteobacteria_Gammaproteobacteria_Aeromonadales_Aeromonadaceae_Aeromonas_Aeromonas.sobria	0.500	0.000	0.001	N
Proteobacteria_Gammaproteobacteria_Aeromonadales_Aeromonadaceae_Aeromonas_Aeromonas.veronii	0.511	0.001	0.001	N
Proteobacteria_Gammaproteobacteria_Enterobacteriales_Enterobacteriaceae_Unknown.genus225_Unknown.species2	0.500	0.000	0.001	N
Proteobacteria_Gammaproteobacteria_Enterobacteriales_Enterobacteriaceae_Enterobacter_Enterobacter.cloacae	0.605	0.015	0.023	N
Proteobacteria_Gammaproteobacteria_Enterobacteriales_Enterobacteriaceae_Escherichia.Shigella_Unknown.species233	0.500	0.000	0.001	N
Proteobacteria_Gammaproteobacteria_Enterobacteriales_Enterobacteriaceae_Escherichia.Shigella_Unknown.species257	0.583	0.008	0.012	N
Proteobacteria_Gammaproteobacteria_Enterobacteriales_Enterobacteriaceae_Escherichia.Shigella_Unknown.species592	0.500	0.000	0.001	N
Proteobacteria_Gammaproteobacteria_Enterobacteriales_Enterobacteriaceae_Serratia_Serratia.fonticola	0.581	0.007	0.011	N
Proteobacteria_Gammaproteobacteria_Pasteurellales_Pasteurellaceae_Haemophilus_Haemophilus.haemolyticus	0.500	0.000	0.001	N
Proteobacteria_Gammaproteobacteria_Pseudomonadales_Pseudomonadaceae_Pseudomonas_Unknown.species591	0.500	0.000	0.001	N
Proteobacteria_Gammaproteobacteria_Pseudomonadales_Pseudomonadaceae_Pseudomonas_Pseudomonas.libanensis	0.500	0.000	0.001	N
Proteobacteria_Gammaproteobacteria_Pseudomonadales_Pseudomonadaceae_Pseudomonas_Pseudomonas.protegens	0.505	0.000	0.001	N
Proteobacteria_Gammaproteobacteria_Pseudomonadales_Pseudomonadaceae_Pseudomonas_Pseudomonas.toyotomiensis	0.500	0.000	0.001	N
Spirochaetes_Spirochaetes_Spirochaetales_Spirochaetaceae_Treponema_Unknown.species745	0.500	0.000	0.001	N
Spirochaetes_Spirochaetes_Spirochaetales_Spirochaetaceae_Treponema_Unknown.species760	0.500	0.000	0.001	N
Spirochaetes_Spirochaetia_Spirochaetales_Brachyspiraceae_Brachyspira_Brachyspira.aalborgi	0.500	0.000	0.001	N
TM7_TM7.3_Unknown.order_Unknown.family_Unknown.genus18_Unknown.species6	0.500	0.000	0.001	N

Table S6. Fitting of colon tissue microbiome datasets to Sloan's (2006, 2007) near-neutral model*

Source community	Destination community	*N*	Immigration Probability (*m*)	R^2	Total Number of Species	Percentage of Species Below Neutral (%)	Percentage of Neutral Species (%)	Percentage of Species Above Neutral (%)
NT	NT	53348	0	-0.687	1749	0.9	94.6	4.5
PT	PT	49187	0	-0.392	1505	1.5	92.3	6.2
NT	PT	49187	0	-0.436	913	2.3	86.3	11.4

*The model-fitting failed in all three cases ($R^2 < 0$).
